# The Impact of Returning Individual Research Results: Program Evaluation of the Michigan Alzheimer’s Disease Research Center’s Participant Feedback Program

**DOI:** 10.1002/alz70861_108817

**Published:** 2025-12-23

**Authors:** Bernardo Flores, Hailey Kohl, Amanda Cook Maher, Betsy Posby, Theresa F. Gierzynski, Gloria Whitaker, Julia Pleskaczynska, Jonathan M Reader, Benjamin M. Hampstead, J. Scott Roberts, Annalise Rahman‐Filipiak

**Affiliations:** ^1^ Michigan Alzheimer's Disease Research Center ‐ University of Michigan, Ann Arbor, MI USA; ^2^ Research Program on Cognition & Neuromodulation Based Interventions ‐ University of Michigan, Ann Arbor, MI USA; ^3^ Michigan Alzheimer's Disease Research Center, Ann Arbor, MI USA; ^4^ Michigan Alzheimer's Disease Research Center‐ University of Michigan, Ann Arbor, MI USA; ^5^ University of Michigan, Ann Arbor, MI USA

## Abstract

**Background:**

Participants in Alzheimer’s disease (AD) studies strongly desire the opportunity to learn their results. However, significant barriers exist to return of results (RoR), including the logistical burdens to studies and potential psychological risks to participants. The Michigan Alzheimer’s Disease Research Center (MADRC) has developed and evaluated the feasibility, effectiveness, acceptability, and safety of RoR to participants in our longitudinal cohort.

**Methods:**

All participants enrolled in the longitudinal cohort complete an annual evaluation including neurologic examination, neuropsychological testing, and clinical interview. A diagnosis is rendered through a consensus meeting using standard clinical criteria. All participants are offered a 1:1 feedback session with a research clinician via phone, video, or in‐person. Participants who complete feedback are also offered the opportunity to take part in the Outcomes Following Feedback on Research Results (OFFeR) study, through which they attend immediate, 1‐week, and 4‐months post‐feedback visits. Acceptability is measured through both feedback uptake and a satisfaction questionnaire. Feedback effectiveness is evaluated through a measure of recall and comprehension of individual results. Safety is measured via the Geriatric Depression Scale‐15 Item (GDS‐15) and the Positive and Negative Affect Scale (PANAS).

**Results:**

As of April 2025, 1,819 RoR visits have been completed, with <5% of UM‐MAP participants declining feedback. Visits were multi‐modal (46% via phone, 37% via video, 4% in‐person, 13% not tracked), occurred an average of 59 days post‐evaluation, and lasted an average of 27 minutes. Among participants enrolled in OFFeR (*n* =263, age=73.87[5.93], education=16.73[2.21], 26% Black/African American or Asian), accuracy of recalled results ranged from 94.6% (immediate) to 84.0% (4‐months). On a 5‐point Likert scale (1=Very Poor/Dissatisfied, 5=Very Well/Satisfied), satisfaction ranged from 4.76 to 4.93 depending on feedback element. GDS‐15 (1‐week=0.93[1.33], 4‐months=0.85[1.08]) and PANAS Negative scores (1‐week=13.75[4.75], 4‐months=13.44[4.3]]) demonstrated minimal distress; there were no adverse events.

**Conclusion:**

Preliminary evidence suggests that the MADRC Feedback Program is feasible and potentially scalable to other Centers and research programs. Participants can understand and recall results, are highly satisfied with their experience, and experience little distress following RoR. RoR may be a strategy for future recruitment and retention of participants in longitudinal Alzheimer's Disease Related Dementias (ADRD) research.

